# Description of a Singular Case of Dropsy of the Periosteum, with a Detachment of the Epiphyses, in a Hydrocephalic Fœtus

**Published:** 1818-03

**Authors:** Jules Cloquet

**Affiliations:** Prosector to the Faculty.


### *For the London Medical and Physical Journal.*

### *Description of a singular Case of Dropsy of the Periosteum, with a detachment of the Epiphyses, in a Hydrocephalic Fœtus;* by JULES CLOQUET, M.D. Prosector to the Faculty.

ON the 22d of August last, the corpse of a male fœtus, of seven months, born the day before, was delivered to Professor Dubois. The mother, twenty-four years old, was of a good constitution, and had observed nothing extraordinary during her pregnancy, except that she had not felt her child for several days before the delivery. After the opening of the membranes, and the escape of the waters, there appeared in the vagina a soft reddish bag, half full of a liquid, in which bony portions could be felt, that seemed to belong to the scull. When the delivery was over, it was evident, that this great bag was formed by the head of a hydrocephalic fœtus, whose limbs, at first sight, appeared to be all fractured. Professor Dubois gave me this fœtus to examine, and I observed as follows. It is fourteen inches long, from the feet to the chin; from the chin to the top of the head, seven inches; whole length, twenty-one inches. The circumference of the head, taken above the orbits, is thirteen inches. The skin, clear and reddish; the epidermis is detached in some parts, without any sign, however, of putrefaction. The head, very capacious, resembles a membranous bag, flattened, flabby, covered with a very fine black hair, but not abundant. Several of the scull-bones may be felt through its sides, which are rather thin, floating in the liquid it contains. Having made a little opening in the upper part, I inflated some air through a tube; the bag became distended, but the air soon escaped through the nostrils, along with a white clotted inodorous liquid. The bag, cut longitudinally, was formed singly of the teguments and pericranium. It contained a transparent liquid rather red, the two portions of the coronal, the two parietal, the superior part of the occipital, and the scaly portion of the left temporal bones.

The dura mater was not distended, but formed another smaller bag, flabby also, situated in the middle of the outer, and half filled by the cerebral substance, softened and almost liquefied; the ethmoid bone was destroyed, and, in its place, was an opening, through which had escaped the cerebral substance, and the serous liquid contained between the dura mater and the pericranium. In fact, the hydrocephalus of this fœtus was external, and the effusion took place immediately round the bones of the cranium. It is easy to convince oneself of this fact, by considering, 1st, That the teguments and pericranium are greatly extended, while the dura mater has preserved its natural size, on which account there is a spacious cavity between those membranes, in which are the bones of the cranium. 2dly, The bones of the scull have not changed their form, nor are broader or thinner, as happens when the serosity breaks into the cavity of the arachnoid membrane, and forms the internal hydrocephalus.

The bones of the face were very moveable in their articulations, principally on the median line. The upper jaw-bones, those of the palate, and the two pieces of the lower jaw-bone are very moveable. The periosteum which covers the facial bones is, in a great part, separated; and, below it, was a serosity more or less copious, which, in some places, raised it up.

All the long bones of the limbs presented a similar separation of the periosteum, with a complete detachment of their epiphyses. The periosteum, very much thickened, was, on each of those bones, like a fibrous bag, very wide,—the extremities of which were closed by the epiphyses; and in the cavity the body of the bone, which floated in the midst of a reddish, diaphanous, viscous, inodorous, and insipid, serosity which distended the bag formed by the periosteum. The body of the bone adhered to this membranous bag only by the principal communicating vessels; the bone was red, porous, and seems to have had life till the moment of the death of the fœtus. Its extremities are separated from the epiphyses by a variable interval, that has as much as two or three lines of extent. The extremities of the bony cylinder, instead of being irregular and granulous, as happens when the epiphyses of a fœtus are separated by a violent effort, or by maceration; are smooth and polished, covered with a soft membrane, red, very tenacious, difficult to separate, and which has the greatest analogy with the sort of false membrane that appears on the extremities of a newly-fractured bone. The correspondent surface of the epiphysis was covered with a similar membrane, but thinner. The separation of the periosteum was completed on the femur, tibia, peroneum, and humerus on each side; that is, the diaphysis of the bone only held to the periosteum by the mere vascular fasciculus, which enters the principal vessels for nourishing the bones. The separation was not general, nor the accumulation of serosity so abundant in the radius, cubitus, or clavicles; the ribs were the same, and it was chiefly towards their sternal extremity that the serosity was accumulated.

The muscles were pale, almost white in some parts; and their insertions adhered strongly to the distended and loosened periosteum. The sort of dislocation of the members of this fœtus, and their extreme mobility in divers points of their extent, are the result of the loosening of all the epiphyses.*

The vertebral column presented nothing particular, except a considerable dilatation of the vertebral canal in the cervical region. The spinal marrow was altered and destroyed near that region; below, it was perfectly sound.

The breast was completely square, as the ribs, being separated from their prolonging cartilages, formed almost right angles.

The ossa coxigis exhibited, in their whole extent, a separation of the periosteum, with a very abundant serous effusion. What appeared particular here was, that the cavity of the periosteum seemed to communicate with the interior of the sacro-iliac articulation, which is extremely moveable; the symphysis pubis was lax, though much less so than the preceding articulation.

The periosteum was not loosened in the metatarsal nor metacarpal bones, nor in those of the tarsus and carpus, which were quite cartilaginous.

The joints of the limbs were formed merely by the loosened epiphyses, and the synovial membrane which lines them was distended by a more abundant synovia than common.

This fœtus presented some further particularities:—

1st. The eyelids were opened by the globe of the eye, which projected considerably, owing chiefly to the disposition of the cornea. This membrane was blackish, almost opaque, pulpy, softish; it was prolonged and projected three lines and a half. It was very thick, but nevertheless hollow,

---

* The limbs were so deformed, that, of several persons to whom I showed the subject, some thought it had an universal dislocation, and others, that they were fractures,—such as Professor Chaussier has related many remarkable instances of. Some, however, suspected a loosening of the epiphyses. I presented this fœtus to the School of Medicine, before I dissected it.

and contained the aqueous humour. This affection of the cornea seemed to form a variety of the staphyloma. The sclerotica was white and very thick; the eye, from before backwards, from the optic nerve to the union of the sclerotica with the cornea, was five lines long, which made the antero-posterior diameter of this organ eight lines and a half; the vertical and transversal diameter being only four lines and a half. The choroid was black, rather thick; retina hardly visible, crystalline, very convex; and the vitreous humour slightly opaque. The sclerotica had within, near its union with the cornea, a beautiful black line, which extended in the form of a little circle.

2dly, The pectoral and abdominal viscera were in their natural state; the peritoneum, however, had a particular alteration worth remarking. Almost the whole of that membrane, which was very thin, was of a light colour, opaque, slightly yellow. At first sight, one would have thought, that some powdery matter had been spread on it, such as carbonate of lead coarsely pounded and diluted in water. In fact, the light colour of the peritoneum was not an uniform tint, but arose from the agglomeration of little white points, a sort of papillæ, separated or united, which rose from the surface of that membrane, to which, in some places, they gave a very fine dead silver colour. This tint was observable, not only on the peritoneum of the sides of the abomen, but also on that which covers the intestines. The epiploon was quite white and opaque. In some places, the papillæ, or white points, formed a membraniform layer, which could be separated, though with difficulty, and which formed several adhesions among the different convolutions of the intestines. I dried, and have preserved, several morsels of peritoneum, covered with this sort of white exudation. One would suppose they were pieces of serous membrane, painted with the chalky half-fluid matter sometimes found in tuberoles.—Was this change owing to an inflammation of the peritoneum?—I cannot decide, and merely relate facts.*

I have preserved several parts of this fœtus, which I intend to deposit among the collections of the faculty.

*Paris; Feb.* 1818.

---

* This alteration of the peritoneum seems peculiar to fœtuses, and is not very uncommon. I have found it several times in fœtuses, whose abdomen was filled with a reddish serosity. In one, this white opaque colour, instead of being spread in large spots, as in the preceding case, was disposed in round distant points, so that the peritoneum seemed all spotted with it. I never met with this peculiar case of pathological alteration in adults; from which, I presume, that, if ever found, it is extremely rare.

